# Supplementary material for: Polysaccharide from Okra (Abelmoschus esculentus (L.) Moench) Improves Antioxidant Capacity via PI3K/AKT Pathways and Nrf2 Translocation in a Type 2 Diabetes Model
Source: Molecules. 2019 May 17;24(10):1906. doi: 10.3390/molecules24101906 (PMC6571734; doi:10.3390/molecules24101906)
Supplement: Supplementary file 1 [file molecules-24-01906-s001.pdf]

molecules

Supplementary material

Article

Polysaccharide from Okra (*Abelmoschus esculentus* (L.) Moench) Improves Antioxidant Capacity via PI3K/AKT Pathways and Nrf2 Translocation in a Type 2 Diabetes Model

Zhengzheng Liao [1], Jingying Zhang [1], Bing Liu [1], Tingxu Yan [2], Fanxing Xu [3], Feng Xiao [2], Bo Wu [2], Kaishun Bi [4], and Ying Jia [2,*]

[1] School of Traditional Chinese Material Medical, Shenyang Pharmaceutical University, Wenhua Road 103, Shenyang 110016, China; Liaozhengzheng1989@163.com (Z.L.); JY1993@163.com (J.Z.); Liubingsy@126.com (B.L.)
[2] School of Functional Food and Wine, Shenyang Pharmaceutical University, Wenhua Road 103, Shenyang 110016, China; Yantingxusyphu@163.com (T.Y.); Fengxiao1996@126.com (F.X.); Bowusyphu@126.com (B.W.)
[3] Wuya College of Innovation, Shenyang Pharmaceutical University, Wenhua Road 103, Shenyang 110016, China; fanxing0011@163.com
[4] School of Pharmacy, Shenyang Pharmaceutical University, Wenhua Road 103, Shenyang 110016, China; kaishunbisyphu@163.com
* Correspondence: jiayingsyphu@126.com; Tel: +86-24-23986933.

2. Results

2.1. OP separation and purification

The Fig.S1A was shown that there are two large elution peaks and named OP-1, OP-2. The same samples were combined. As a result, the higher yields of OP-2 was for further purified using an open Sepharose CL -6B column and the outflow curve was shown in Fig.S1B, and the fraction named OP.

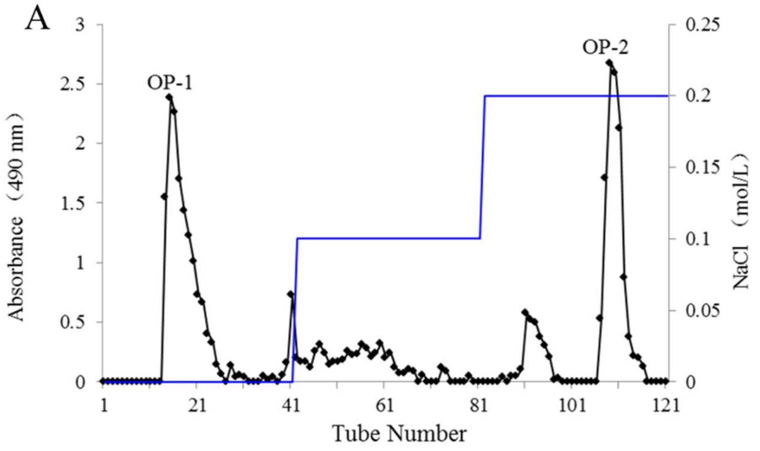

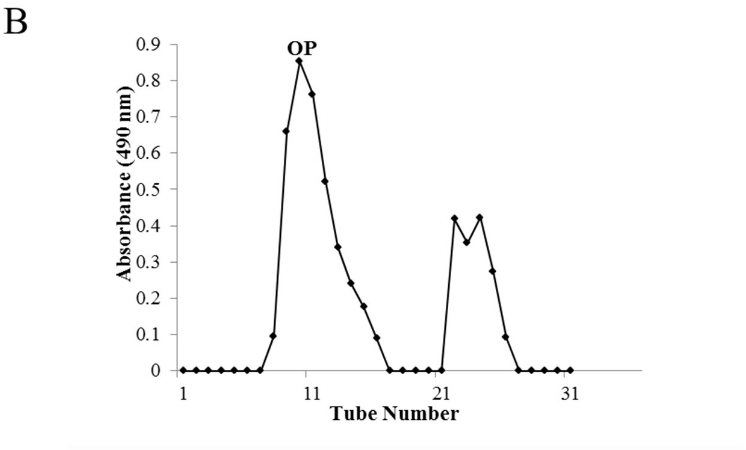

Fig. S 1. Purification curve of OP on DEAE-Sepharose Fast Flow and Sepharose CL-6B gel column. (A) Elution profile of okra crude polysaccharide from DEAE-Sepharose Fast Flow column. (B) Elution profile of sub-fraction OP-2 eluted from Sepharose CL-6B gel column.

2.2. FT-IR spectrum analysis of OP

As shown in Fig.S2, an intense and broad band at around 3422 cm^{-1} was the O-H stretching vibration and approximately 2923 and 2852 cm^{-1} corresponded to the C-H stretching vibration. The absorption peaks at 1614 cm^{-1} was the C=O asymmetrical stretching of -COO [1]. Two absorbance peaks at 1439 and 1401 cm^{-1} was attributed to C-O stretching and C-H bending, indicating the presence of pectin methyl ester group (-OCH_3) [2]. The band at 1270 cm^{-1} associated with the –S=O stretching vibration, which shows the existence of sulphate group [3], while signals at 1370 cm^{-1} represent araboxylan [4]. Particular polysaccharide has specific bands in the range 1200-1000 cm^{-1} region, and this region is dominated by ring vibrations overlapped with stretching vibrations of (C-OH) side groups and the (C-O-C) glycosidic band vibration. The absorption at 1172, 1103, 1051, 1006, 615 and 420 cm^{-1} were dominated by a pyranose ring in polysaccharides [4].

p

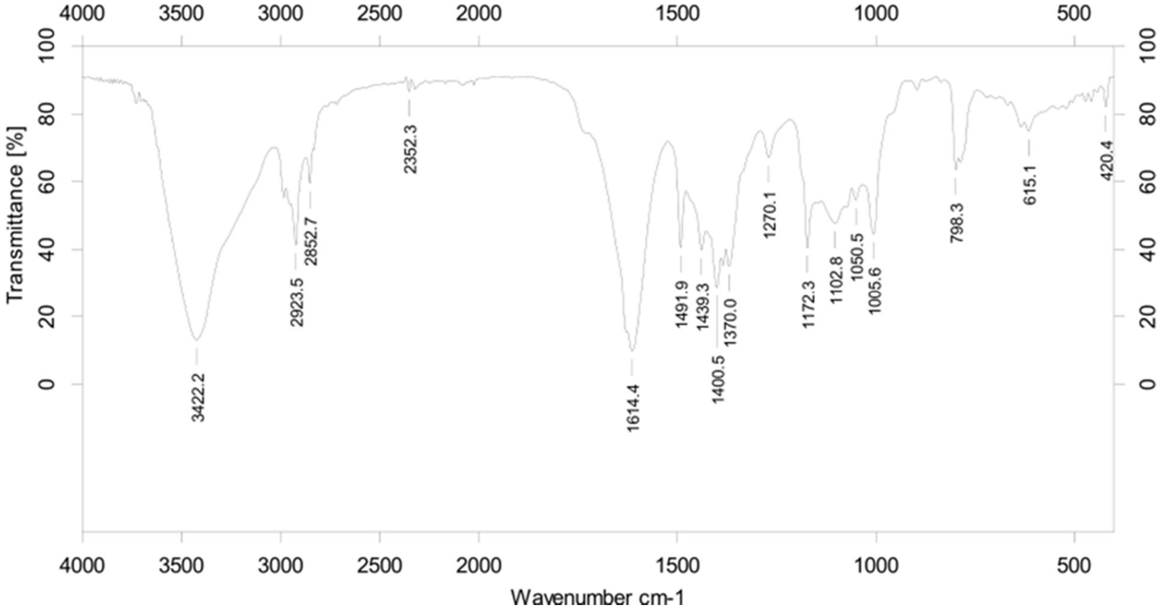

Fig. S 2. The FT-IR spectra of OP.

2.3. Monosaccharide component analysis of OP

Fig.S3 shown the chromatographic profile of OP revealed that monosaccharide component of OP by analysis was the mannose, rhamnose, glucuronic acid, galactosal acid, galactose and arabia, the molar ratio was 3.4:3.76:24.19:6.27:8.73:3.13.

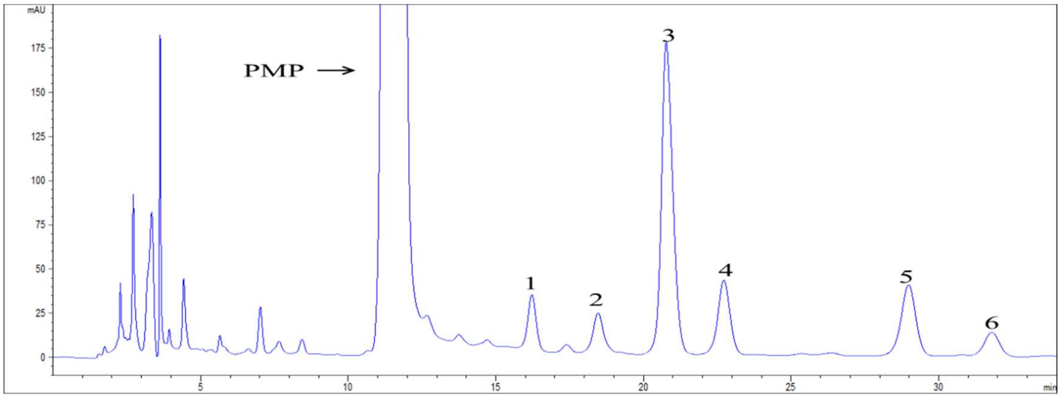

Fig. S 3. Finger analysis of OP by RP-HPLC. (1) Mannose (2) Rhamnose (3) Glucuronic acid (4) Galactosal acid (5) Galactose (6) Arabia.

4. Materials and Methods

Table S 1. Nutritional composition of the high fat diet fed to mice.

Content[a]	Diet (g/kg)
Casein	25.85%
Cystine	0.39%
Maltodextrin	16.15%
sucrose	8.89%
Cellulose	6.46%
Soybean oil	3.23%
lard	31.66%
Mine-rich M1002	0.79%
Calcium hydrogen phosphate	0.68%
Calcium carbonate	0.71%
Tartaric acid choline	2.13%
Multidimensional V1001	1.29%
choline bitartrate	0.26%
cholesterol	2.00%

[a] The crow diet has energy of 5.24 kcal/g, with 20% carbohydrates, 60% fat and 20% protein. The details of ingredient composition of the high fat diet were provided by the company.

FT-IR spectroscopy analysis

Fourier transform infrared (FT-IR) spectroscopy was carried out by using a Bruker EQUINOX55 infrared spectrometer (Bruker Company, Germany). The OP (1 mg) was in KBr powder and pressed into pellets and the spectra recorded with at wavelengths of 4000 to 400 cm^{-1}.

References

1. Zheng, W.; Zhao, T.; Feng, W.W.; Wang, W.; Zou, Y.; Zheng, D.H.; Takase, M.; Li, Q.; Wu, H.Y.; Yang, L.Q.; Wu, X.Y. Purification, characterization and immunomodulating activity of a polysaccharide from flowers of *Abelmoschus esculentus*. *Carbohyd. Polym.* 2014, 106, 335-34.
2. Wu, M.B.; Zhang, F.F.; Yu, Z.P.; Lin, J.P.; Yang, L.R. Chemical characterization and in vitro antitumor activity of a single-component polysaccharide from *Taxus chinensis* var. *mairei*. *Carbohyd. Polym.* 2015, 133, 294-301.
3. Zhang, L.; Tu, Z.C.; Wang, H.; Kou, Y.; Wen, Q.H.; Fu, Z.F.; Chang, H.X. Response surface optimization and

physicochemical properties of polysaccharides from *Nelumbo nucifera* leaves. *Int. J. Biol. Macromol.* 2015, 74, 103-110.

4. Wang, K.L.; Li, M.; Wen, X.; Chen, X.S.; He, Z.Y.; Ni, Y.Y. Optimization of ultrasound-assisted extraction of okra (*Abelmoschus esculentus* (L.) Moench) polysaccharides based on response surface methodology and antioxidant activity. *Int. J. Biol. Macromol.* 2018, 114, 103-110.

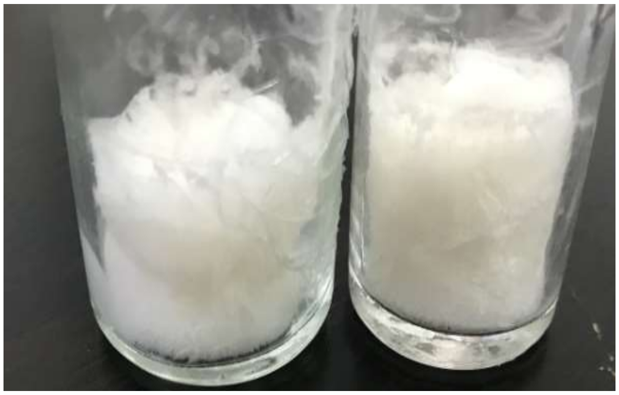

Fig. S 4. The shape of OP in the vial.
